# Crystal structure of human METTL6, the m^3^C methyltransferase

**DOI:** 10.1038/s42003-021-02890-9

**Published:** 2021-12-03

**Authors:** Ran Chen, Jie Zhou, Ling Liu, Xue-Ling Mao, Xiaolong Zhou, Wei Xie

**Affiliations:** 1 MOE Key Laboratory of Gene Function and Regulation, State Key Laboratory for Biocontrol, School of Life Sciences, The Sun Yat-Sen University, Guangzhou, Guangdong 510006 People’s Republic of China; 2 Key Laboratory of Tropical Marine Bio-resources and Ecology, Guangdong Key Laboratory of Marine Materia Medica, Innovation Academy of South China Sea Ecology and Environmental Engineering, South China Sea Institute of Oceanology, Chinese Academy of Sciences, No. 1119, Haibin Road, Nansha District, Guangzhou, 511458 People’s Republic of China; 3 State Key Laboratory of Molecular Biology, CAS Center for Excellence in Molecular Cell Science, Shanghai Institute of Biochemistry and Cell Biology, Chinese Academy of Sciences, University of Chinese Academy of Sciences, 320 Yue Yang Road, Shanghai, 200031 People’s Republic of China

**Keywords:** Transferases, X-ray crystallography

## Abstract

In tRNA, the epigenetic m^3^C modification at position 32 in the anticodon loop is highly conserved in eukaryotes, which maintains the folding and basepairing functions of the anticodon. However, the responsible enzymes METTL2 and METTL6 were identified only in recent years. The loss of human METTL6 (hMETTL6) affects the translational process and proteostasis in cells, while in mESCs cells, it leads to defective pluripotency potential. Despite its important functions, the catalytic mechanism of the C32 methylation by this enzyme is poorly understood. Here we present the 1.9 Å high-resolution crystal structure of hMETTL6 bound by SAH. The key residues interacting with the ligand were identified and their roles were confirmed by ITC. We generated a docking model for the hMETTL6-SAH-CMP ternary complex. Interestingly, the CMP molecule binds into a cavity in a positive patch with the base ring pointing to the inside, suggesting a flipped-base mechanism for methylation. We further generated a model for the quaternary complex with tRNA^Ser^ as a component, which reasonably explained the biochemical behaviors of hMETTL6. Taken together, our crystallographic and biochemical studies provide important insight into the molecular recognition mechanism by METTL6 and may aid in the METTL-based rational drug design in the future.

## Abstract (web summary)

Ran Chen et al. report the crystal structure of human m3C methyltrasfease METTL6. They generate structure models of hMETTL6 in complex with substrates, providing important insight into the molecular recognition mechanism by METTL6 and may aid in the METTL-based rational drug design in the future.

## Introduction

Epigenetic modifications on nucleic acids play crucial roles in gene regulation^1^. All known RNA species are subjected to chemical modifications, among which transfer RNA (tRNA) is the most extensively modified type^2^. The modifications affect the folding, stabilities, and biological functions of tRNA^3,4^. For instance, the prevalent modifications in the anticodon loop of tRNA promote translation efficiencies by maintaining the conformation of the anticodon loop, enhancing codon-anticodon interactions, and preventing frameshifting, etc^5–11^. Additionally, these modifications may mediate other critical biological and physiological processes as several lines of evidence indicate that defects in many modifications in humans are associated with the pathogenesis of various cancers, underscoring the pivotal roles of tRNA modifications in organismal physiology and fitness^4^.

To date, more than 170 different types of RNA modification have been identified^12^, as a result of the recent development of more sensitive and quantitative technologies. Methylation is most common, and it can be catalyzed by a large family of methyltransferase-like proteins (METTLs) that transfer the methyl group from S-adenosylmethionine (SAM) to a variety of positions in RNA nucleosides^13–16^. Multiple METTL proteins, including METTL3 and METTL14, have been well characterized, which produce the 6-methyladenosine (m^6^A) modification in mRNA, long intergenic ncRNA (lincRNA), and microRNA (miRNA), etc.^17,18^.

The 3-methylcytosine (m^3^C) modification is present in both tRNA and mRNA and displays diverse roles in developing diseases through the regulation of tRNA fate^19^. In tRNA, the m^3^C modification at position 32 in the anticodon loop is highly conserved in eukaryotes, which maintains the folding and basepairing functions of the anticodon^20^. The key enzyme discovered first is TRM140 from *Saccharomyces cerevisiae*, specific to tRNA^Thr^ and tRNA^Ser^ methylation. In contrast, in the fission yeast *Schizosaccharomyces pombe*, two ScTrm140 homologs encoded by the *Trm140* and *Trm141* genes are collectively responsible for the m^3^C production in tRNA^Thr^ and tRNA^Ser^^16,21^. As to humans, the homologs of Trm140 and Trm141 encoded by the *METTL2A*, *METTL2B*, *METTL6*, and *METTL8* genes have been identified by sequence similarity analyses^15,16^. Specifically, METTL2A and METTL2B are required for the threonyl and argininyl tRNA methylation, while METTL8 is for mRNA. The expression levels of METTL8 in most breast cancers are upregulated, mediated by the transcription factor Yin Yang 1 (YY1), which in turn modify AT-rich interactive domain-containing protein 1 A (ARID1A) to promote tumor growth and the migration of cancer cells^22^. On the other hand, METTL6 is a tRNA^Ser^-specific m^3^C methyltransferase, whose knockdown would substantially reduce the susceptibility of lung cancer cells to cisplatin^23^. In highly proliferative cells in patients with breast cancer, the high expression levels of METTL6 are usually correlated to bad prognosis^24^. A study showed that METTL6 catalyzes the methylation of C32 in several tRNA^Ser^ isoacceptors^25^. It enhances the proliferative activity of hepatocellular carcinoma (HCC) by affecting the relevant genes involved in multiple cellular processes including cell cycles, apoptosis, stemness, and also maintains the self-renewal potential in mESCs cells^25^. These studies demonstrated that METTL6 plays critical role in tumorigenesis and the development, invasion as well as susceptibility to drugs. Recently, we reconstituted the METTL2A and METTL6 methylation systems in vitro and demonstrated that both enzymes need particular prerequisites for the m^3^C32 modification^26^. Specifically, the anticodon loop and the long variable arm of tRNA^Ser(GCU)^ are key determinants for its C32 methylation, which is also critically dependent on the presence of both hMETTL6 and hSerRS^26^. An aminoacylation-defective SerRS mutant is able to stimulate the methylation activity of hMETTL6 to a comparable level of wild-type (WT) SerRS, suggesting that SerRS contributes to the methylation through tRNA binding. However, the cooperative mechanism among hMETTL6, hSerRS, and tRNASer(GCU) remains to be further explored.

In this study, we solved the crystal structure of human METTL6 in complex with S-adenosyl-L-homocysteine (SAH) at 1.9 Å and characterized the association mode between the enzyme and the cofactor. By structural comparison and modeling studies, we analyzed the possible binding mode of the cytosine 5'-monophosphate (CMP) and tRNA molecules and proposed a model for the catalytic pathway, which may provide fundamental insight into the catalytic mechanism and possible conformational changes of paralogous enzymes such as METTL2 and METTL8 and their roles in anti-cancer therapies.

## Results

### The overall structure of human METTL6

**Fig. 1:**
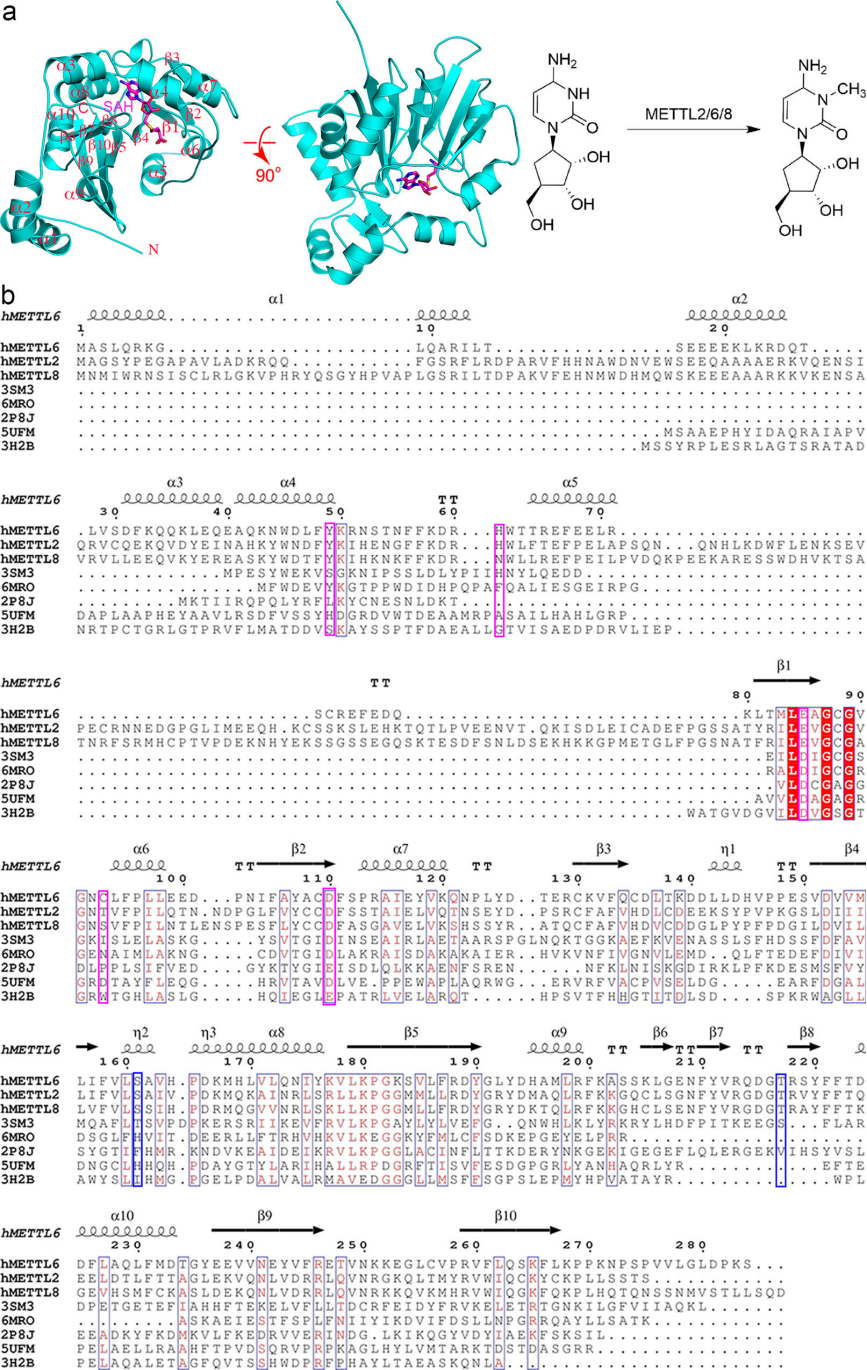
The overall structure of hMETTL6. **a** The chemical reaction that hMETTL6 catalyzes and its structural basis. Left: the structure was shown in cartoon in two orthogonal views and the SAH ligand was shown as sticks. The N- and C-termini were indicated, and the helices were labeled. Right: the chemical reaction carried out by the METTL2/6/8 enzymes. **b** Multiple sequence alignment of hMETTL6 with orthologs and other published structures. The secondary structure elements were labeled above the sequences. The key residues for SAH contacts were indicated by the red arrows. The magenta boxes indicated the residues important for SAH binding, whereas blue boxes indicated the residues important for CMP binding.

To find out the molecular mechanism of human METTL6, we solved the structure of hMETTL6 in complex with SAH at a high resolution. The sample for crystallization contained the uncut N-terminal tag. The final model ultimately resolved the full-length protein (H-10-P273) except for an internal disordered fragment (T248-C256, Table 1). The final model contains 276 residues in total. The enzyme exhibits a typical Rossmann fold (Fig. 1a). The N-terminus starts with a long loop formed by the affinity tag, followed by a helix (α1). The α2 helical region (S16-D25) is highly flexible and is mainly composed of polar residues, which display poor density. Therefore, the side chains of most residues of α2 were not modeled. The helices flanked each side of the central β-sheet formed by seven strands. Of these, the β10-strand is antiparallel to all the other strands. The sequence alignment is shown in Fig. 1b and the topology of the protein is shown in Supplementary Fig. 1. In addition to the 174 water molecules in the model, a SAH molecule is bound at the active site (Table 1).

**Table 1:** Data collection and refinement statistics.

	hMETTL6
*Data collection*	
Space group	*P23*
Cell dimensions	
*a*, *b*, *c* (Å)	110.26, 110.26, 110.26
*α*, *β*, *γ* (°)	90.00, 90.00, 90.00
Resolution (Å)	50–1.90 (1.97–1.90)^a^
*R*_merge_	0.19 (1.76)
*I/σΙ*	28.0 (2.0)
Completeness (%)	100 (100)
Redundancy	38.5 (27.4)
*Refinement*	
Resolution (Å)	25.30–1.90 (1.95–1.90)
No. reflections	35334
*R*_work_/*R*_free_	0.179 (0.201)
No. atoms	
Protein	2209
Ligand/ion	26
Water	174
B-factors	
Protein	36.9
Ligand/ion	22.8
Water	40.0
R.m.s. deviations	
Bond lengths (Å)	0.008
Bond angles (°)	1.13

^a^Values in parentheses are for highest-resolution shell. Statistics belong to one crystal.

### The SAH binding mode

**Fig. 2:**
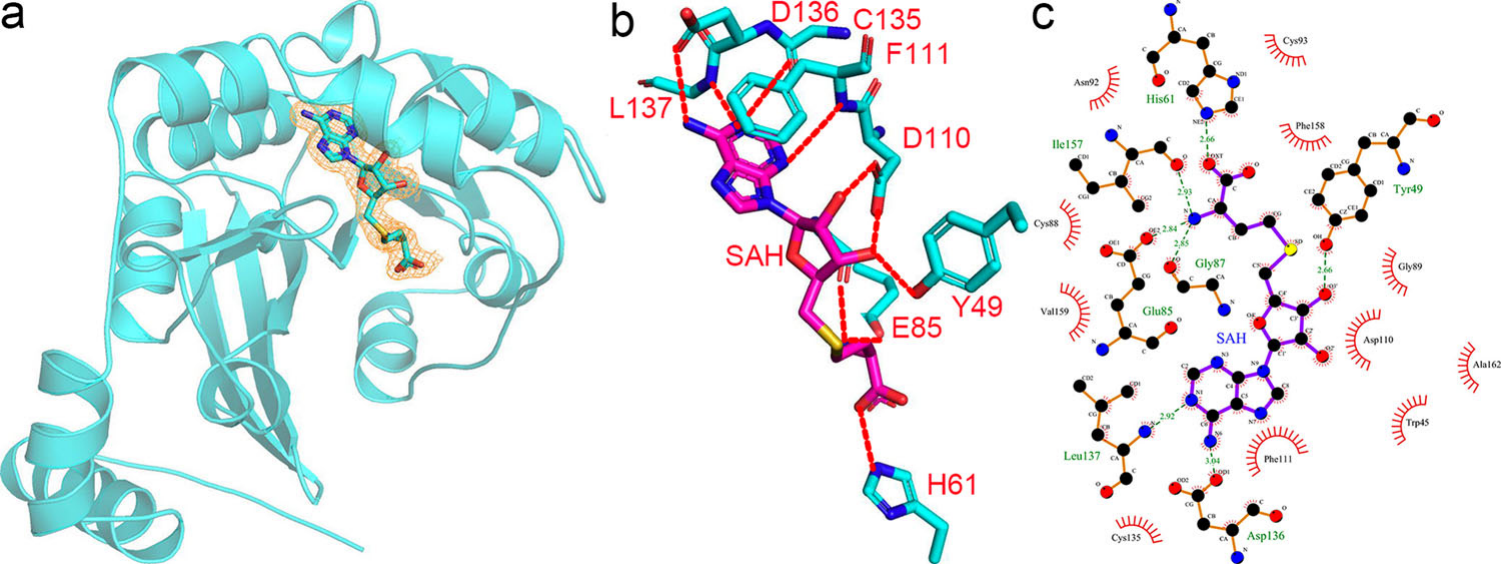
The interaction network of SAH bound by hMETTL6. **a** The composite omit map was countered at 2σ and indicated by the orange mesh. **b** The interaction network that SAH participates in hMETTL6. The dashed lines indicated hydrogen bonds. **c** The ligplot showing the interactions of SAH with the active site residues.

The SAH ligand fits snuggly in the binding pocket with well-covered density (Fig. 2a), fixed by the interactions with the enzyme, including hydrogen bonds, salt bridges, and hydrophobic interactions. Specifically, the adenine ring makes multiple hydrogen bonds with the backbone nitrogen of L137, C135, F111, and the terminal carboxyl group of D136, respectively. Additionally, it stacks onto the aromatic ring of F111. The ribose ring also forms three additional hydrogen bonds using its 2′- and 3′- hydroxyl groups, with the side chains of D110 and Y49. Finally, the carboxyl group of SAH accepts a hydrogen bond from H61 while it donates three to G87, I157, and E85, respectively, including some backbone interactions (Fig. 2b, c).

**Fig. 3:**
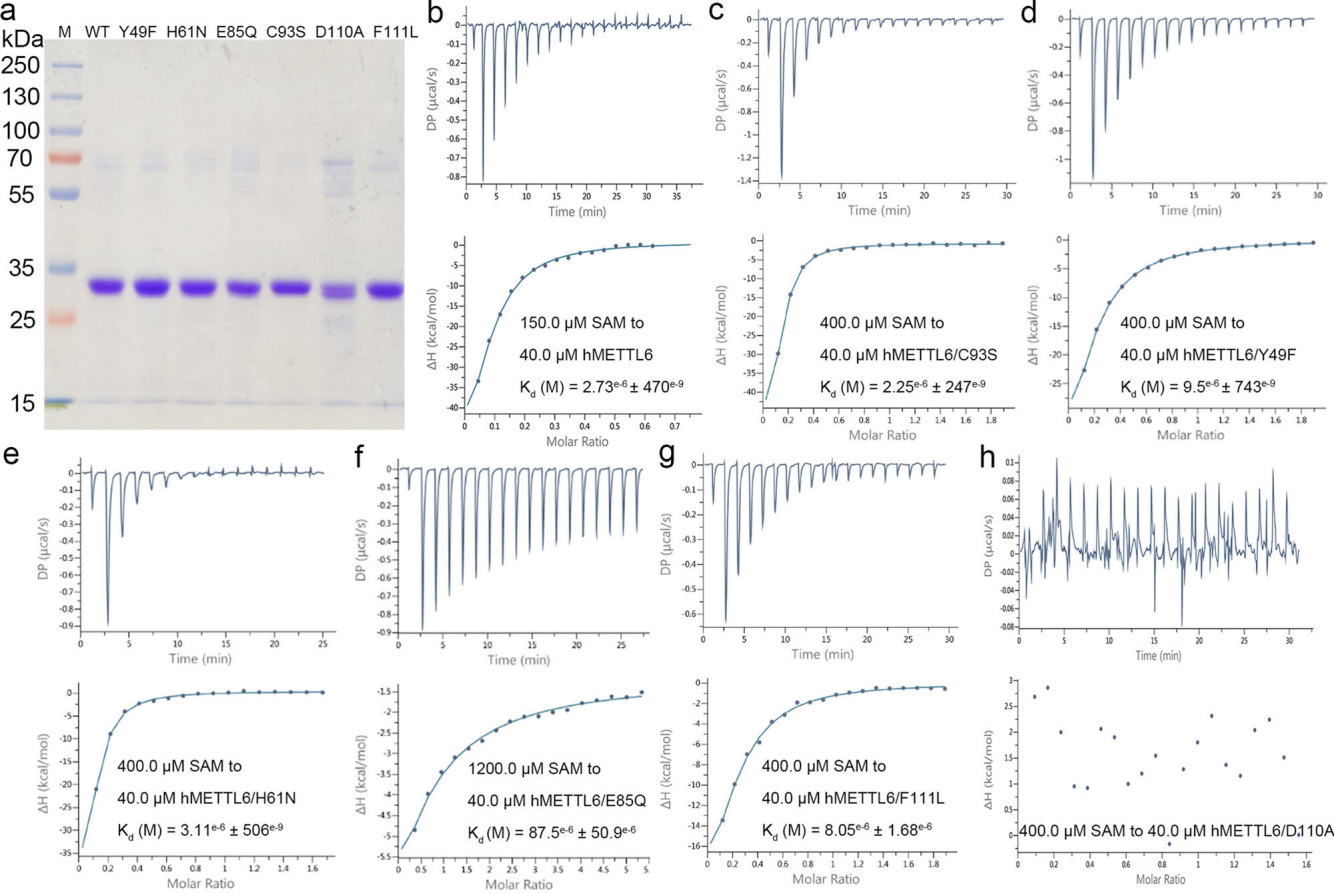
The ITC titration of hMETTL6 WT and mutants by SAM. **a** The purity assessment of the hMETTL6 WT and mutants by SDS-PAGE gel electrophoresis. **b**−**h** The titration curves of hMETTL6 WT and mutants by SAM. The final *K*_D_ values were given for all the mutants.

Therefore, we mutated these residues (Y49F, H61N, E85Q, C93S, D110A, and F111L) to evaluate their contribution to SAH binding. These mutants were well expressed and purified to homogeneity (Fig. 3a) and we checked the folding states of each mutant by size-exclusion chromatography (Supplementary Fig. 2). The results showed that E85Q and D110A would form aggregation to certain extents, with the latter mutation greatly disrupting the structural integrity of the protein, while the rest mutants maintained their folding states. We measured the affinity of these mutants to SAM by isothermal titration calorimetry (ITC). The affinity of WT enzyme to SAM is in the micromolar range (~2.7 μM), and the C93S mutation barely changed the affinity (Fig. 3b, c). However, the Y49F, H61N and F111L mutations approximately increased the *K*_D_ value by ~3−4 folds (Fig. 3d, e, g), suggesting that they play greater roles in the binding of SAM. Lastly, the E85Q mutation reduced the affinity by more than 30-fold (Fig. 3f), while the D110A mutation almost eliminated the association capacity of the enzyme (Fig. 3h). This result could be partially explained by the unfolding of the protein, but it also indicated that these two residues play vital roles in the interactions with the cofactor because some portions of the mutants still remained in their native states.

### The oligomeric state

**Fig. 4:**
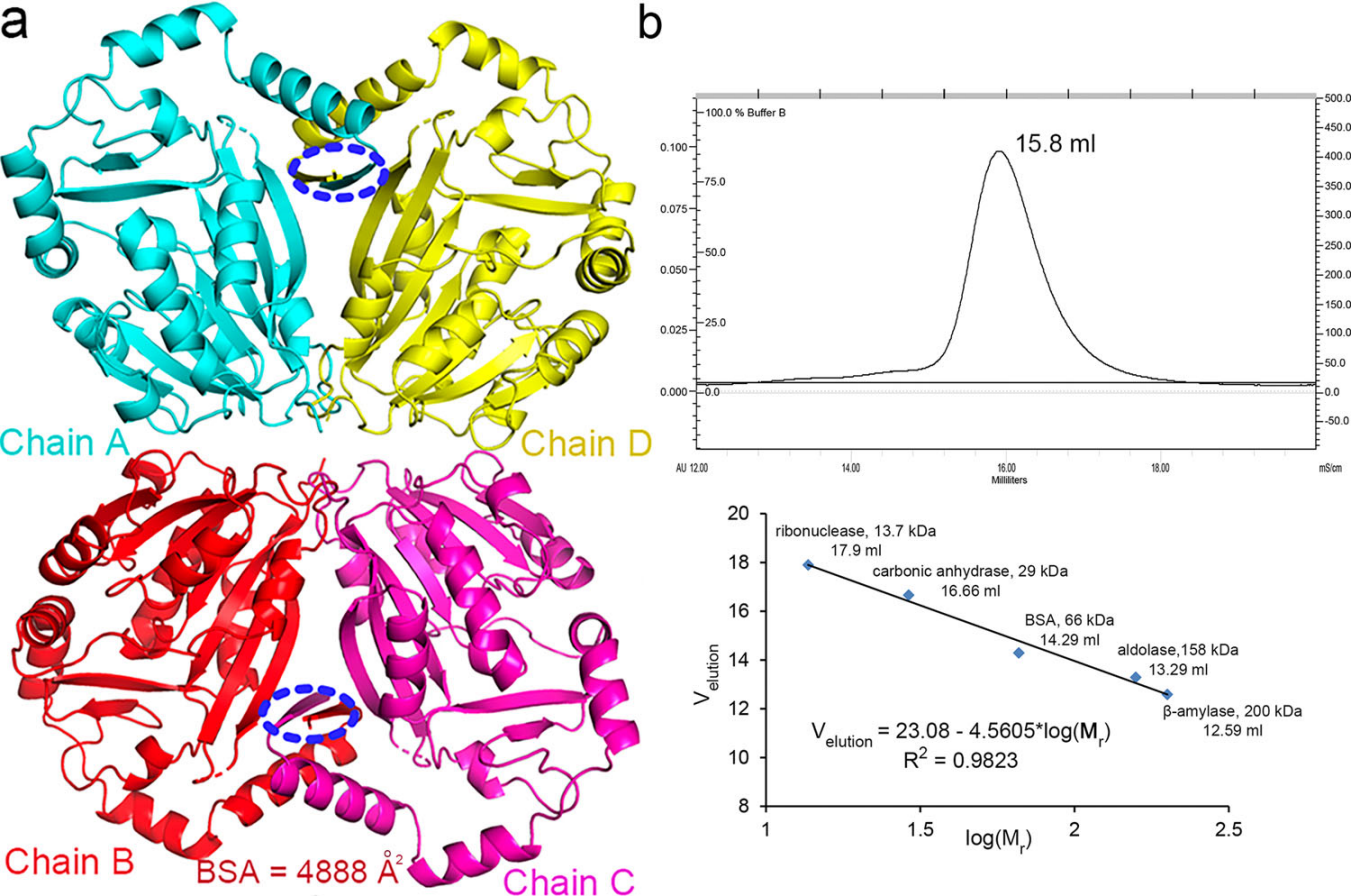
The oligomeric state analysis. **a** The crystal packing pattern of hMETTL6. The four subunits were colored differently and shown in cartoon. BSA: buried surface area. **b** The size exclusion chromatography analysis of the oligomeric state of hMETTL6. Top: the elution profile of hMETTL6; Bottom: the elution profile of the molecular standards. The equation describing the relationship between the retention volumes and their corresponding molecular masses was shown below the curve.

Although there is only one molecule in the asymmetric unit, the PDBePISA webserver suggested that hMETTL6 forms a tetramer (Fig. 4a). The calculated buried surface area between chains A and B is 1004 and 4888 Å^2^ between chains A and D, respectively. In contrast, the buried surface area between chains A and C is only 424 Å^2^. Therefore, at least chains A and D share a large interface and raise the possibility of them being a dimer. Notably, the interface is potentially mediated by the N-terminal affinity tags involving mainly hydrophobic interactions and hydrogen bonds (residues H-1-G-3), which form a pair of antiparallel strands. To validate this theory, we performed size exclusion chromatography analysis. The protein elutes at 15.8 ml on a Superdex 200 column (Fig. 4b), suggesting a molecular weight of ~34.6 kDa and therefore still a monomer in solution (calculated molecular weight is ~35.7 kDa). Taken together, the protein remains monomeric in solution and the tetrameric form is only due to crystal packing.

### Possible CMP binding mode and validation

**Fig. 5:**
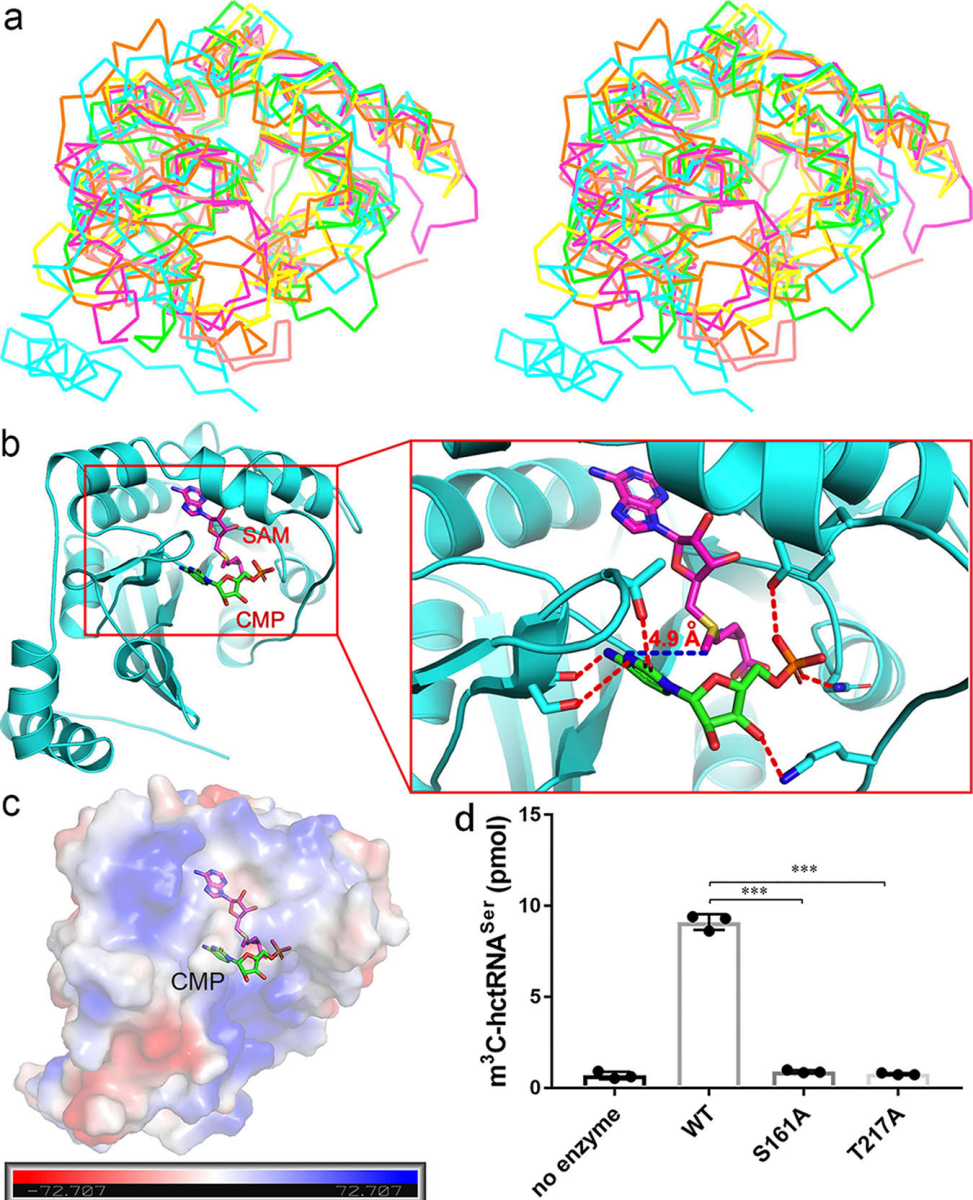
The structural superposition and possible CMP binding mode. **a** structural superimposition of the backbone structure of hMETTL6 (PDB 7EZG, colored cyan) onto that of SAM-dependent methyltransferase cg3271 from *Corynebacterium glutamicum* (PDB 3H2B, green), SAM-dependent methyltransferases q8puk2_metma from *Methanosarcina mazei* (PDB 3SM3, magenta), methyl transferase from *Methanosarcina acetivorans* (PDB 6MRO, yellow), SAM-dependent methyltransferase (np_349143.1) from *Clostridium acetobutylicum* (PDB 2P8J, pink), and *Burkholderia thailandensis* 1,6- didemethyltoxoflavin-N1-methyltransferase bound by 1,6-didemethyltoxoflavin and SAH (PDB 5UFM, orange). The figure was shown in cross-eyed stereoview. **b** The docking model of hMETTL6-SAM-CMP ternary complex. Right: the close-up of the active center. The 4.9 Å distance between the methyl group carbon of SAH and N1 of CMP was indicated by the blue line. **c** The surface representation of the complex. **d** The methyltransferase activity assays of the two mutants S161A and T217A. The WT activity was normalized to 100%, while the activities of the mutants were shown as percentages of that of WT. Data are shown as mean ± SD. Each group was compared using un-paired *t*-test (*n* = 3; two-tailed *P* value; ***P < 0.0001).

The structure of SAH-bound hMETTL6 was searched against the Protein Data Bank for possible evolutionary insight. Most of the hits among the list were methyltransferases, but the relevant studies of most proteins were not published. Among these, the 1,6- didemethyltoxoflavin-n1-methyltransferase from *Burkholderia thailandensis* (named BthII1283 hereafter) complexed with an inhibitor (1,6-didemethyltoxoflavin) and SAH (PDB 5UFM)^27^ caught our attention. BthII1283 catalyzes a single methyl transfer to N1 of 1,6-didesmethyltoxoflavin (1,6-DDMT) in vitro. When superposed with hMETTL6, their Cα traces could be aligned with an RMSD of 1.97 Å over 224 Cαs (Fig. 5a). We found that while the SAH positions in the two complexes could be well superimposed, the involved residues from their respective proteins are not conserved.

To elucidate the enzyme recognition on cytosine, we tried the crystallization of cytosine or CMP into the active site. However, we failed to obtain the cocrystals. ITC showed that the SAH-bound hMETTL6 (40 μM) was never fully titrated by cytosine at a concentration in 50-fold excess, suggesting poor binding affinity of hMETTL6 to a single nucleobase. The enzyme probably requires an entire tRNA molecule to increase the interactions. Therefore, we docked the ligand CMP into the pocket. The identification of the binding site was performed with Autodock vina (ver. 1.5.6), and the grid box was set with the dimensions at ∼20–40 Å. The protein pre-bound with the SAM cofactor was used as the receptor, where the SAM molecule was taken from PDB 3H2B and was manually superimposed onto that of SAH-bound hMETTL6. CMP was the ligand. The potential binding modes generated by the program were further evaluated by the following criteria: (1) The scoring function gave by the program. The CMP molecule should establish specific interactions (hydrogen bonds) with the enzyme; (2) N3 of the CMP molecule should be close enough to allow the methyl group to be transferred; (3) to distinguish cytosine from uracil, N4 of cytosine should be recognized by the enzyme. Based on these principles, we selected the most plausible model out of nine generated by the program. Coincidentally, it was approximately located to the same site as 1,6-didemethyltoxoflavin when the hMETTL6 protein structure was superimposed onto that of BthII1283 (PDB 5UFM), as shown by Fig. 5b. In the model generated, CMP sits above SAM and well fits into the pocket. In this ternary complex, the CMP molecule binds a positively charged cavity with the base ring points to the catalytic center, consistent with the base-flipping mechanism (Fig. 5c). The methyl group is 4.9 Å from N3, while N4 and O2 each form a hydrogen bond with the side chain of S161 and T217, respectively. The phosphate group also makes a hydrogen bond with the side chain of Y49 and N92 respectively, while O3’ of the ribose interacts with K58. Despite the same binding site, the cytosine ring tilts and forms an angle of ~45° from the purine ring of the inhibitor 1,6-DDMT. Y190 of METTL6 poses steric clashes with the planar ring of 1,6-DDMT, and therefore the cytosine ring rotates an angle to avoid these clashes (Supplementary Fig. 3). Due to the predicated importance of the critical residues, we made the S161A and T217A mutations and tested their impacts on the methylation activity in the presence of human SerRS (hSerRS). These two mutants tended to form aggregates, and we could observe species of higher molecular weights on the SDS-denaturing gel upon purification (Supplementary Fig. 4). Our results also showed that the two mutants almost wholly lost their activities (similar to the reaction background where no enzyme was added (Fig. 5d), which partially supported our docking model.

### Model of the quaternary complex and implications

**Fig. 6:**
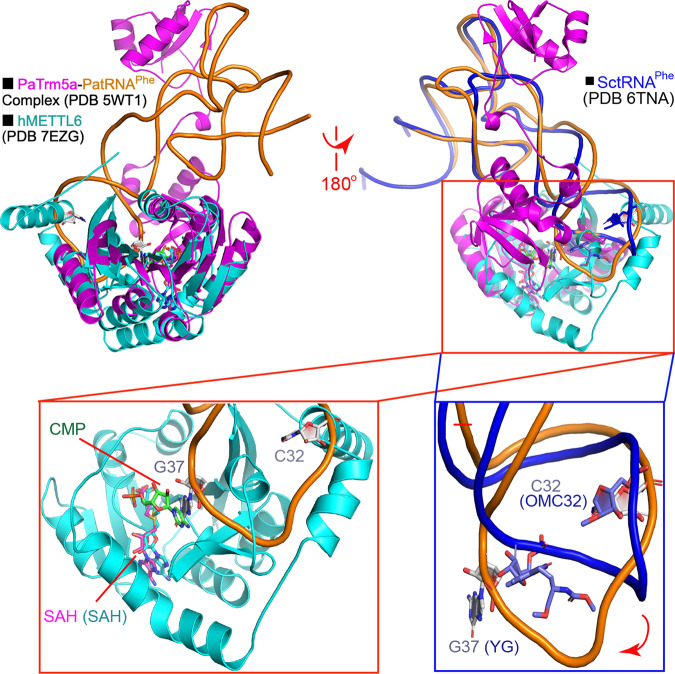
The binary model of the hMETTL6-tRNA complex. Top panel: the superimposition of hMETTL6 with that of the PaTrm5a-PatRNA^Phe^-SAH complex in the front and back views (PDB 5WT1). The blue molecule on the top right corner represents the yeast tRNA^Phe^ molecule (SctRNA^Phe^) with a classical conformation (PDB 6TNA). Bottom panel: the close views of the anticodon loop interactions with the hMETTL6-SAH complex (left) and the comparison of these regions from two tRNA molecules (right). The red arrow indicated the rotation of the anticodon loop needed for the methylation. The bases at positions of 32 and 37 were shown. Note that the bases of SctRNA^Phe^ were modified. OMC: 2′-O methylcytosine; YG: wybutosine. The coloring of the molecules corresponded to their names.

The success of the docking model motivated us to explore the possible binding modes of tRNA and hSerRS as well, through multiple superimpositions. First, we superimposed the structure of our hMETTL6 complexed by the CMP molecule (PDB 7EZG) onto that of Trm5a in complex with its cognate substrate tRNA^Phe^ and SAH (PDB 5WT1)^28^, using the “SSM Superpose” command in *Coot*^29^ to further study the recognition mechanism. Trm5a from the archaeon *Pyrococcus abyssi* (PaTrm5a) displays bifunctional methylation activities (including the m^1^G37 production capability in tRNA^Phe^) and is a crucial enzyme for the G37 hypermodification in archaea. Surprisingly, although it does not share considerable sequence homology with hMETTL6, parts of the structures (especially of the methyltransferase domains or the Rossmann domain) were aligned well (Fig. 6). Their Cα traces could be superimposed with an RMSD of 2.8 Å over 142 Cαs. Moreover, the SAH molecules from the two PDB files were bound at almost identical sites. CMP (the green molecule in Fig. 6) was bound at a similar location to that of G37, and the two nitrogen atoms to be methylated (located at equivalent positions of the six-membered rings of their respective bases) were only separated by 3.6 Å, but the phosphate groups pointed to opposite directions. The structural discrepancies between here and aforementioned suggested that the nucleotide phosphate CMP may arrange itself for the optimal chance to be methylated. On the other hand, the conformation of tRNA^Phe^ bound by PaTrm5a is distorted, which unwinds its loop region upon the interactions with the enzyme^28^, as the generic tRNA^Phe^ molecule shows a distinct local structure (PDB 6TNA)^30^. The deviations here further imply the local reorganization of the tRNA loop, which needs to undergo a rotation similar to that displayed by the PaTrm5a enzyme upon the methylation reaction^28^. Furthermore, the region flanking α9 have visible structural clashes with the anticodon loop and would likely rearrange themselves as well.

**Fig. 7:**
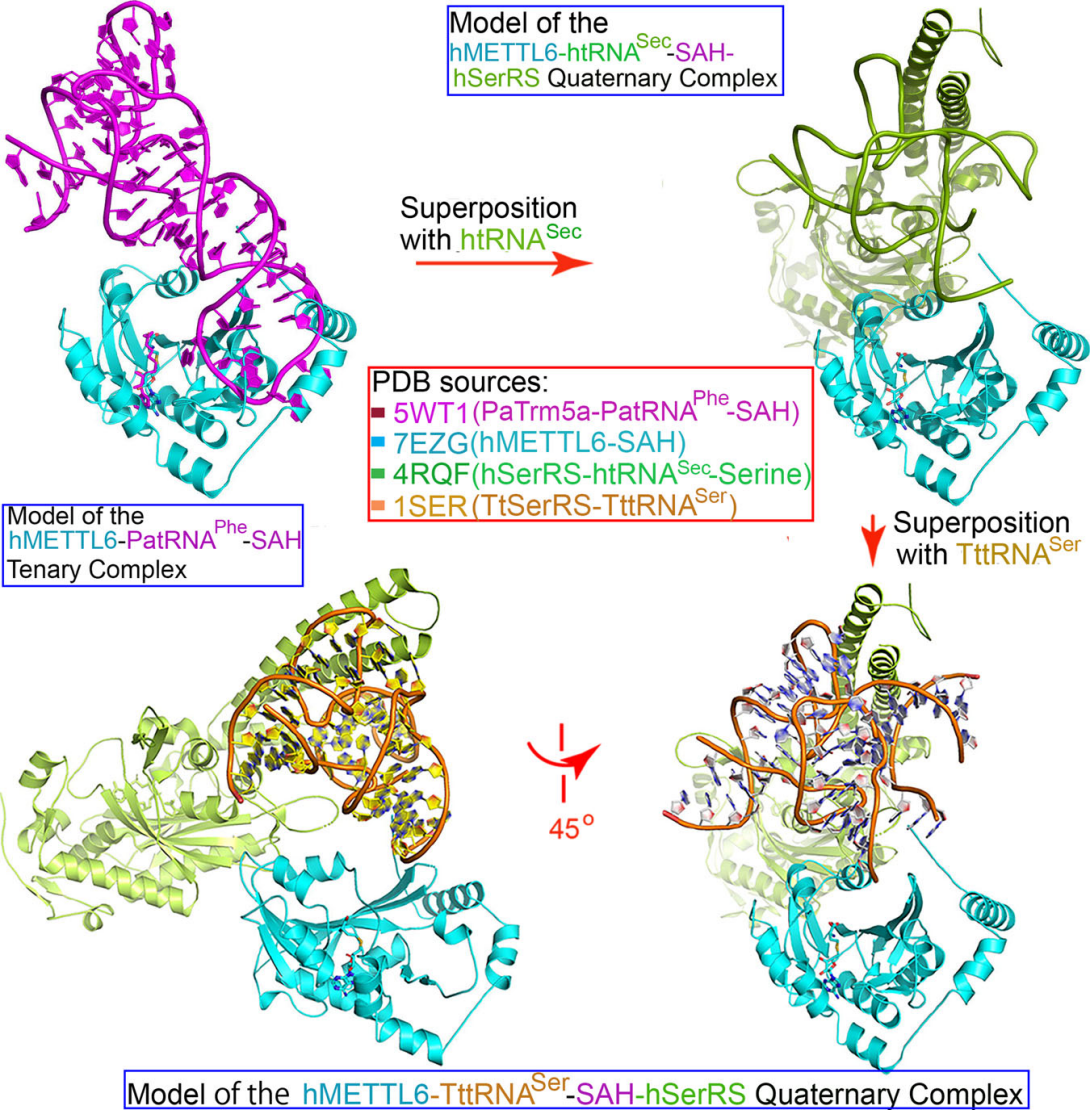
The model generation of the hMETTL6-tRNASer-hSerRS-SAH quaternary complex. The starting point, the hMETTL6-PatRNA^Phe^-SAH complex was obtained from Fig. 5. The protein and tRNA molecules were colored coded, which match the colors of the PDB codes given in the central box. Pa: *Pyrococcus abyssi*; Tt: *Thermus thermophilus*.

Next, we laid the backbone of the tRNA^Sec^ molecule complexed by hSerRS (PDB 4RQF) onto that of tRNA^Phe^ using the “LSQ Superpose” command in *Coot*^29^ to generate the model of the hMETTL6-tRNA^Sec^-SAH-hSerRS complex, followed by the superposition and replacement of the tRNA^Sec^ molecule by the *Thermus thermophilus* tRNA^Ser^, whose anticodon loop is disordered in the structure (PDB 1SER)^31^. Thus, our hMETTL6-tRNA^Ser^-hSerRS model was completed (Fig. 7). The two protein molecules bind distinct regions of tRNA^Ser^ (the long variable arm and anticodon loop, respectively), but they do not display steric hindrances. Additionally, both studies^15,26^ have suggested a tRNA-dependent interaction between hSerRS and hMETTL6; thus, these possible interactions might be insufficient for a stable complex in the absence of tRNA, which awaits further experimental verification.

## Discussion

The m^3^C modification was discovered about fifty years ago, but its physiological significance was unclear because the responsible enzymes and their natural substrates were never identified until recently. Compared to the well-characterized m^6^A modification and the structure of the METTL3-METTL14 complex, the m^3^C modification mechanism is poorly understood. Three enzymes, METTL2, METTL6, and METTL8, so-called “writers”, catalyze the methyl transfer to cytosine in tRNA and mRNA, respectively. In this study, we solved the structure of hMETTL6, which to the best of our knowledge, is the first structure of the m^3^C methyltransferase. While it displays a common Rossmann fold, hMETTL6 shows features that distinguish itself from other transferases. The closest structural homolog (PDB 3H2B) could be aligned with an RMSD larger than 1.6 Å (over 166 Cα atoms), suggesting functional differences.

In our cocrystal structure, SAH binds snuggly at the active site of hMETTL6, and the binding mode was confirmed by our ITC titrations. However, we did not obtain the cocrystals for the single base of cytosine or CMP, which probably binds with a much lower affinity than that of SAH. However, our docking model shows that residues K58, S161, T217, and S219 may interact with the cytosine base to be methylated. The surface charge rendering of the apo-protein showed negatively charged cavities and patches suitable for tRNA binding. Interestingly, the CMP molecule binds into a cavity on the surface of the protein with the base ring pointing to the inside, suggesting a flipped-base mechanism for methylation. In our subsequent modeling studies, we obtained a model for the hMETTL6-tRNA^Ser^-hSerRS-SAH quaternary complex, with each macromolecule occupying an angle of the tRNA triangle. Additionally, we predict that the anticodon loop, including C32 and the Y190-A202 region of hMETTL6, would undergo relatively large conformational changes to rotate and flip the base into the methylation pocket. Nevertheless, the accurate model necessitates the cocrystal structure of the hMETTL6-tRNA-SAM/SAH-SerRS quaternary complex.

The significant sequence homologies between METTL2/6/8 suggest that the three proteins share a common catalytic mechanism, supported by the conservation of the critical residues. Hence, the results we obtained from hMETTL6 may also apply to the other two methyltransferases as well. For example, hMETTL2A was also found to be monomeric in solution, as was the case for hMETTL6. On the other hand, hMETTL2 displays a unique N-terminal extension compared to hMETTL6, which may contribute to the differential recognition efficiencies toward various tRNA species.

Lastly, METTL6 has been recently demonstrated to affect gene expression, cell homeostasis, tumor cell growth, etc. As a potential new oncogene, *METTL6* would represent a promising drug target and this study may provide the structural basis for future rational design against relevant cancers.

## Methods

### Cloning, expression, and purification of the proteins

The wild-type (WT) full-length hMETTL6 gene (GenBank Accession No. NM_152396) was amplified from cDNA library of the human 293T cells, which encodes 284 residues. It was cloned into the expression vector pET-28a (+) vector (Novagen, Germany) with the restriction sites *NdeI* and *XhoI*, in which an 8 × His-tag and a PreScission protease cleavage site were placed immediately upstream of the multiple cloning site (MCS). The mutants were generated by the *QuikChange* method (Agilent, USA) using the WT gene as the template. All the plasmids were transformed into *E. coli* strain BL21 (DE3) cells, and the cells were cultured in Luria-Bertani broth containing 30 μg ml^−1^ kanamycin at 37 °C. The overexpression was induced by 0.3 mM isopropyl *β*-_D_−1-thiogalactopyranoside (IPTG) when the OD_600_ value reached 0.6−0.8 and was kept shaking overnight at 25 °C. The *E. coli* cells were then harvested by centrifugation at 3030 × *g* for 20 min and resuspended in pre-cooled nickel-nitrilotriacetic acid (Ni-NTA) buffer A (20 mM Tris-HCl (pH 8.0), 250 mM NaCl, 10 mM imidazole, 1 mM *β*-mercaptoethanol (*β*-ME), and 1 mM PMSF. The cells were disrupted by ultrasonication, and the supernatant was obtained by centrifugation at 23,500 × *g* for 1 h at 4 °C. The supernatant was then applied to a Ni-NTA affinity column (Qiagen, Germany), which was previously equilibrated with Ni-NTA buffer A. The target protein was eluted with Ni-NTA buffer B (20 mM Tris-HCl (pH 8.0), 250 mM NaCl, 250 mM imidazole, 1 mM *β-*ME, and 1 mM PMSF). The hMETTL6-containing fractions were pooled, dialyzed in a buffer containing 20 mM Tris-HCl (pH 8.0), 50 mM NaCl and 1 mM DTT. hMETTL6 was further purified by anion exchange chromatography with a Q-HP column (GE Healthcare, USA) with a NaCl gradient. The pure protein was collected and concentrated to 4 mg/ml. The aliquoted protein was flash-frozen and stored at −80 °C. For mutants to be tested with aminoacylation activity assays, 5% glycerol was added to the concentrated protein before it was frozen.

### Crystallization, data collection, and structure determination

The initial screens for hMETTL6 crystals were manually set up using the sitting-drop vapor-diffusion method, with the Crystal I and II, PEGRx, and Index screens (Hampton Research, USA). The protein sample was mixed with 2 mM SAH and incubated for 10 min. The sample of the complex was centrifuged at 23,500 × *g* for 10 min prior to crystallization. Hits were observed two weeks later, small triangle-shaped crystals were obtained, generated in conditions containing ammonium sulfate. After optimization, small triangle-shaped crystals were obtained from 2 M ammonium sulfate, 0.2 M potassium sodium tartrate tetrahydrate, 0.1 M sodium citrate tribasic dehydrate (pH 5.6). The fully-grown crystals were soaked in a freshly made cryoprotective solution containing all the components of the reservoir solution plus 20% (v/v) glycerol. The soaked crystals were mounted on nylon loops and flash-cooled in liquid nitrogen.

A 1.9 Å diffraction dataset was collected using beamline19U (BL19U) at the Shanghai Synchrotron Radiation Facility (SSRF, Shanghai, P. R. China)^32^ and was processed with the program *HKL3000*^33^. The structure was solved by molecular replacement using the program *PHENIX* with the coordinates of 5F8C as the search model. The initial model was based on the solution and built manually according to the electron density map with *COOT*^29^. Multiple cycles of refinement alternating with model rebuilding were carried out by *PHENIX.refine*^34^. The final model was validated by *molprobity*^35^, with 97.79, 2.21, and 0% of the residues falling into the favored, allowed, and disallowed regions in the Ramachandran plot. The structural figures were produced with *PyMOL* (www.pymol.org). All data collection and refinement statistics are presented in Table 1.

### ITC measurements

ITC experiments were conducted at 25 °C using a PEAQ ITC titration calorimeter (Malvern instruments, UK). To exactly match their buffer compositions, the hMETTL6 proteins (WT or mutants) were dialyzed against the same buffer containing 20 mM Tris-HCl (pH 8.0), 150 mM NaCl, and 1 mM DTT. The affinities were measured by titrating SAM (150.0 μM) against WT hMETTL6 (40.0 μM), or SAM (400.0 μM) against hMETTL6 mutants (40.0 μM). The first injection of 0.4 μL was followed by 16–18 injections of 2 μL drops. The MICROCAL ORIGIN software was used to determine the site-binding models that produced good fits. Individual peaks from titrations were integrated and displayed on a Wiseman plot. The first reading was removed from the analysis. The binding affinity (*K*_D_) and change in enthalpy (Δ*H*) associated with the binding events were calculated after fitting the dataset.

### Methyltransferase activity assays

The assays followed the same procedure as described^26^. The reaction mixes were incubated for 5 min before the reactions were stopped. Each variant (WT or mutants) was assayed three times independently.

### Gel filtration chromatography

The molecular size of the hMETTL6 was determined by gel filtration chromatography using a Superdex 200 column (10/300, GE Healthcare, USA) equilibrated with 20 mM Tris-HCl buffer (pH 8.0) containing 150 mM NaCl and 1 mM DTT. The column was calibrated with the marker proteins consisting of ribonuclease (13.7 kDa), carbonic anhydrase (29 kDa), bovine serum albumin (BSA) (66 kDa), aldolase (158 kDa), and β-amylase (200 kDa) to generate the calibration curve. The retention volume of hMETTL6 was compared with the markers to estimate the apparent molecular weight.

### Statistics and reproducibility

General data analysis (means and standard deviation) was performed primarily by Prism 7.0. All experiments were performed with biological triplicates and values were expressed as means ± standard errors.

### Reporting summary

Further information on research design is available in the Nature Research Reporting Summary linked to this article.

## Supplementary information

Supplementary Information

Description of Additional Supplementary Files

Supplementary Data 1

Reporting Summary

## Data Availability

The structure was deposited in the PDB, accession number 7EZG. The uncropped gel images were shown as Supplementary Figs. 5 and 6. The source data is given as Supplementary Data 1. Any remaining data is available from the corresponding authors upon reasonable request.
