# Supplementary material for: Crystal structure of human METTL6, the m3C methyltransferase
Source: Commun Biol. 2021 Dec 3;4:1361. doi: 10.1038/s42003-021-02890-9 (PMC8642396; doi:10.1038/s42003-021-02890-9)
Supplement: Supplementary file 1 — Supplementary Information [file 42003_2021_2890_MOESM1_ESM.pdf]

Supplementary Information

Crystal structure of human METTL6, the m^3C methyltransferase

Ran Chen[1,2], Jie Zhou[1], Ling Liu[1], Xue-Ling Mao[3], Xiaolong Zhou[3] & Wei Xie [1*]

[1] MOE Key Laboratory of Gene Function and Regulation, State Key Laboratory for Biocontrol, School of Life Sciences, The Sun Yat-Sen University, Guangzhou, Guangdong, 510006, People's Republic of China

[2] Key Laboratory of Tropical Marine Bio-resources and Ecology, Guangdong Key Laboratory of Marine Materia Medica, Innovation Academy of South China Sea Ecology and Environmental Engineering, South China Sea Institute of Oceanology, Chinese Academy of Sciences, No.1119, Haibin Road, Nansha District, Guangzhou 511458, People's Republic of China

[3] State Key Laboratory of Molecular Biology, CAS Center for Excellence in Molecular Cell Science, Shanghai Institute of Biochemistry and Cell Biology, Chinese Academy of Sciences, University of Chinese Academy of Sciences, 320 Yue Yang Road, Shanghai 200031, People's Republic of China

Correspondence to Wei Xie: xiewei6@mail.sysu.edu.cn

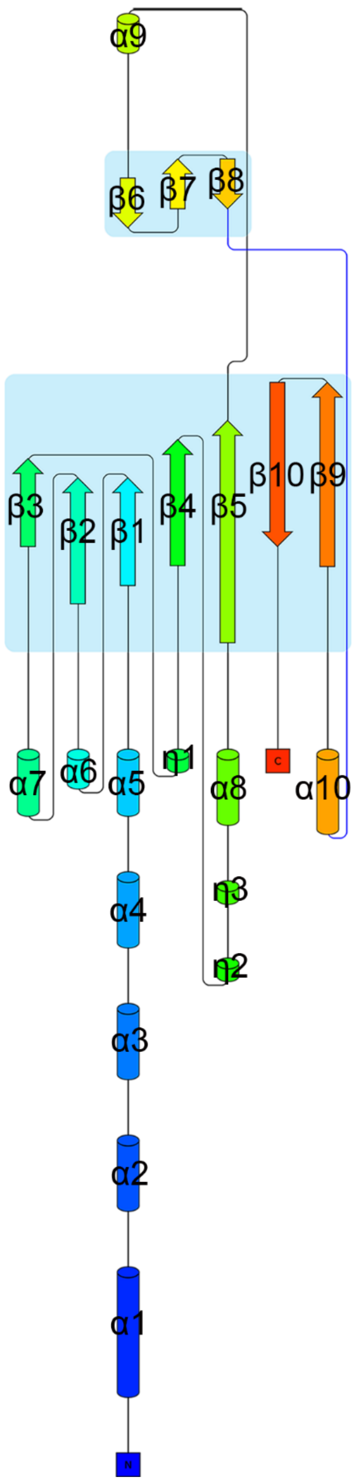

Supplementary Figure 1. Topology of hMETTL6 generated by the program Pro-origama. The colors change from cold to warm as the sequence proceeds from the N- to the C-terminus.

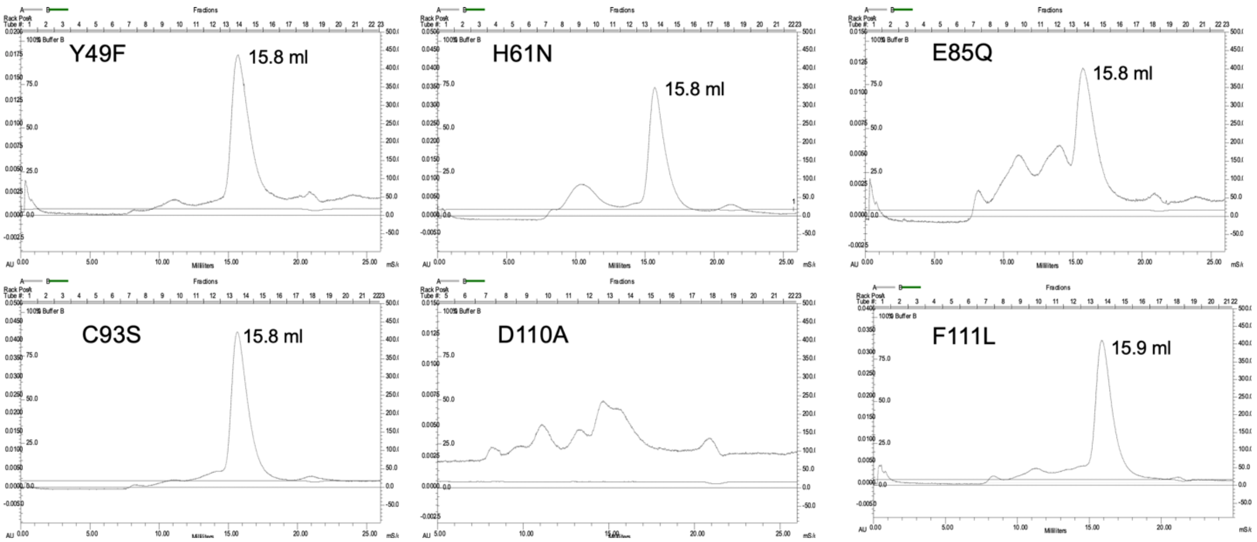

Supplementary Figure 2. The analyses of the folding states of the mutants by size exclusion chromatography. The name and the retention volume of the major peak for each mutant were labeled.

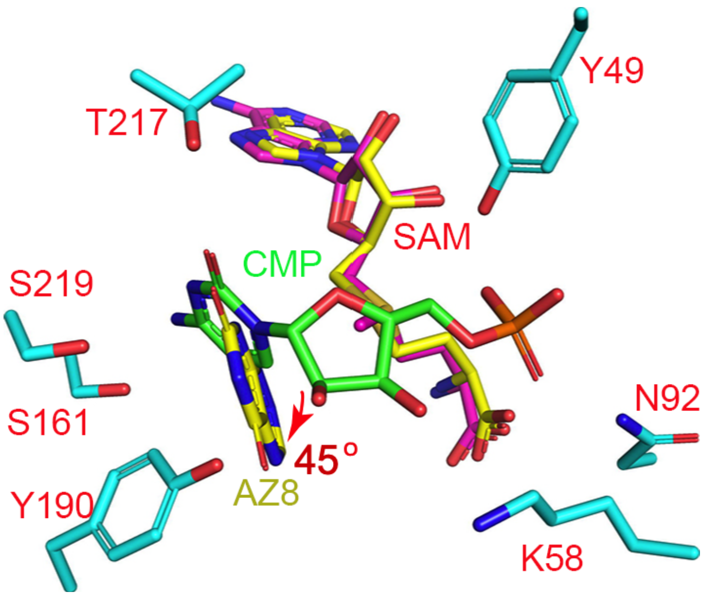

Supplementary Figure 3. The superimposition of the active site of the hMETTL6-SAM-CMP complex with that of BthII1283-SAH-1,6-didemethyltoxoflavin (AZ8) complex. CMP, SAM, AZ8 and SAH were colored green, magenta, yellow and yellow, respectively.

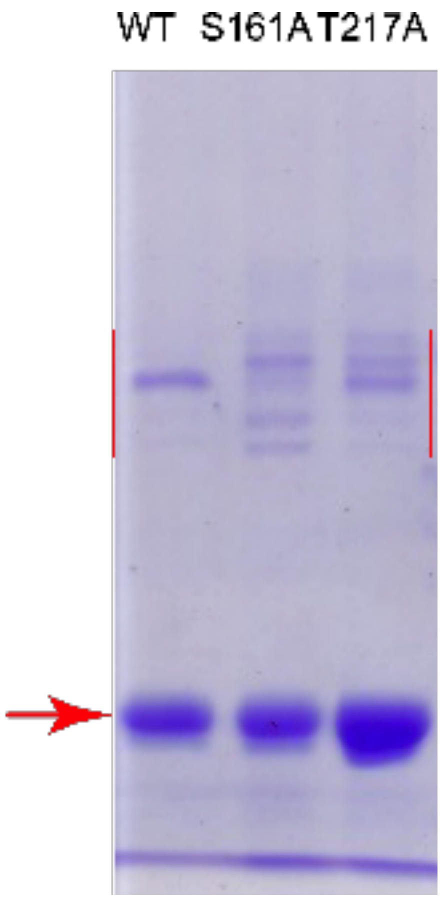

Supplementary Figure 4. The purity assessment of the three mutants by the reducing SDS-PAGE gel electrophoresis. The red arrow indicated the desired band for each variant and the bands in the red box represented aggregated species.
